# Barriers and facilitators to adoption and use of fuel pellets and improved cookstoves in urban Rwanda

**DOI:** 10.1371/journal.pone.0203775

**Published:** 2018-10-08

**Authors:** Ryan Seguin, Valerie L. Flax, Pamela Jagger

**Affiliations:** 1 UNC Project–Malawi, Lilongwe, Malawi; 2 RTI International, Research Triangle Park, NC, United States of America; 3 Department of Public Policy, University of North Carolina, Chapel Hill, NC, United States of America; 4 Carolina Population Center, University of North Carolina, Chapel Hill, NC, United States of America; University of Sydney, AUSTRALIA

## Abstract

**Background:**

The environmental and health impacts of reliance on solid fuels and traditional cookstoves in low-income countries have motivated the promotion of household cooking energy systems that use cleaner burning fuels and cookstoves that lead to reduced exposure to harmful pollutants. Little is known about adoption and use of such systems from the users’ perspective.

**Methods:**

We explored the facilitators and barriers to adoption and use of a private sector marketed household cooking energy system that uses sustainably produced biomass pellets and the cleanest burning fan micro-gasification stove currently available. We conducted 48 in-depth qualitative interviews in Gisenyi, Rwanda with decision-makers and cooks in 16 households that adopted the improved cookstove system and 8 non-adopter households.

**Results:**

Reported facilitators and barriers to adoption and non-adoption, as well as use and non-use were complex, and in some cases, contradictory. Some adopters noted that cleanliness and low smoke production were major facilitators to adoption and use, while other adopters and non-adopters said the cookstoves blackened and damaged cooking pots and produced excessive smoke. Our findings suggest that correct use of the stove mediates user experience. Cost was likewise reported as a facilitator among some adopters and a barrier among other adopters and non-adopters. Peer influence played a significant role as both a barrier and a facilitator to adoption and transcended other factors. Positive peer influence describing the cleanliness, affordability, and efficiency of the cookstove system encouraged adoption and use, while negative comments by peers regarding excessive smoke and damaged cooking pots discouraged adoption. Commentaries by some participants suggest that inadequate training and instruction may be primary causes of the discrepancies.

**Conclusion:**

Cost, cleanliness, communication among peer networks, and adequate training and instruction are important factors associated with the adoption and use of improved cookstoves and should be prioritized in the implementation of improved cookstove programs.

## Introduction

More than 2.7 billion people worldwide depend on solid fuels, including firewood, charcoal, crop residues, and animal dung for cooking. Solid fuels are commonly used in low-income countries. Throughout sub-Saharan Africa, more than 80% of the population relies on them. In Rwanda, 98% of the population uses solid fuels, representing the highest proportion globally [1]. In addition to being inefficient and unsustainable sources of energy, solid fuels produce harmful emissions, leading to household air pollution (HAP). More than four million people die prematurely each year from exposure to HAP caused by solid fuels. Women and children are especially vulnerable because they are frequently exposed to significant levels of HAP for several hours each day [2]. Exposure to HAP accounts for more than half of premature deaths due to pneumonia among children under five years of age [2]. Switching to cleaner, more efficient cooking fuels and technologies is hypothesized to bring about the greatest reduction in HAP [3]. Correct usage of improved cookstoves (ICS) and their prescribed fuels may lower HAP levels by improving combustion efficiency, reducing smoke production, and decreasing exposure to harmful emissions through accelerated cooking time.

Although clean-burning fuels and ICS are effective means of addressing HAP and its related health impacts, there are several potential barriers to switching from traditional fuels and stoves, including cost, infrastructure, technology, information, and socio-cultural factors [4,5]. In many cases, clean-burning fuels are more expensive than traditional fuels and infrastructure for local production and access to a sustained supply of cleaner fuels can be inadequate. New features associated with ICS (e.g., relative to the three-stone fire or basic charcoal stove, many ICS utilize features that regulate temperature, air flow etc.), coupled with inadequate information regarding their use, can also hinder adoption. Additionally, socio-cultural factors can be significant barriers to adoption as cultural norms related to food and cooking practices play a crucial role in many of the communities that rely on traditional cooking fuels and stoves [4,6]. While general factors impeding adoption of cleaner fuels and ICS are known, there is little information on barriers and facilitators to adoption and use of clean fuels from the perspective of community members. Such information is needed to plan programs to expand use of cleaner fuels and reduce HAP exposure in low-income countries.

The aim of this study is to describe barriers and facilitators to adoption and use of a new household energy system comprised of a clean-burning fuel (e.g., densified biomass pellets) and a high performing ICS among household decision-makers and cooks in urban Rwanda. The study was conducted in a community where 95% of households use purchased charcoal as their baseline fuel and where Inyenyeri, a for-profit firm operates. Inyenyeri is a Rwandan social benefit company that leases an ISO Tier 4 fan micro-gasificationi cookstove, the Mimi Moto, to households for a one-time fee of 5,000 Rwandan francs (approximately US$6) coupled with an annual payment contract for a monthly supply of sustainably produced fuel pellets. The quantity of fuel pellets specified in the contract is based on set weight increments and prices, ranging from 30 to 60 kilograms and 4,000 to 8,000 Rwandan francs. Households learn about Inyenyeri through marketing campaigns to groups of villages (i.e. Cells), village cooking demonstrations, and door to door visits from Inyenyeri Customer Service Representatives. Marketing messages include: “cook fast”, “stay clean”, “life made easy”, and “always the cheapest fuel”. Inyenyeri staff deliver fuel pellets to customers’ homes monthly, and customers can purchase additional fuel pellets as needed. Inyenyeri strives for a high level of customer support offering various resources, including free maintenance and repairs, and checking in with customers on a regular basis.

Charcoal and relatively simple fixed or portable charcoal stoves are the baseline fuel/technology for >90 percent of households in our sample. Inyenyeri’s biomass fuel pellets are priced competitively with traditional fuels. The fair pricing aspect of the marketing model mitigates potential cost and infrastructure barriers and allows for in-depth exploration of less-understood implementation barriers by removing the influence of relative cost of adoption from household decision making.

The use of charcoal or firewood (non-clean burning solid fuels) and traditional cookstoves is highly prevalent in Rwanda; therefore, understanding barriers to adoption and use of clean fuels in this setting may be particularly relevant to other countries with limited ICS adoption. Our study is also relevant because it takes place in a medium-sized urban center in Rwanda. As population growth and urbanization take place at very high rates in Africa, understanding drivers and barriers to adoption in such settings may facilitate development of effective strategies for scaling up interventions.

## Methods

### Design

This paper presents the qualitative findings from a sub-study of a larger randomized controlled trial on the health impacts of adoption of Inyenyeri’s household energy cooking system [7]. We conducted in-depth interviews with main decision-makers and main cooks in households that had adopted ICS and those that had not. Main decision makers are those primarily responsible for determining household purchases and who were identified by household members as the person responsible for making decisions about signing a contract with Inyenyeri. The main cook is the person who does the majority of the cooking for the household. Main decision makers and cooks were eligible to be interviewed for this study if they met the above criteria and were ≥ 18 years of age. Cookstove adopters were defined as households that had signed a contract with Inyenyeri in the past year; non-adopters had been marketed to by Inyenyeri, but elected not to sign a contract.

Data collection took place in Gisenyi, Rwanda in July 2016 and was conducted by the University of North Carolina in collaboration with Laterite, a research firm based in Rwanda. The Institutional Review Board at the University of North Carolina, the Rwanda National Ethics Committee, and National Institute of Statistics of Rwanda provided ethical approval for the study. Gisenyi, in Rubavu District, Northwestern Rwanda is the country’s third largest city. It was chosen as the launch site for Inyenyeri due 30% lower overhead than the capital city of Kigali, proximity to poor households, and to the dense urban city of Goma, Democratic Republic of Congo (just 1 km from Inyenyeri’s pilot pellet production factory), which represents a potential future market for the firm.

### Sample

We selected households that had participated in the quantitative baseline data collection in 2015 and to which the firm had marketed ICS and fuel pellets. Our sample size was based on the principle of saturation in qualitative research, which is the point at which additional interviews provide no new information and data collection is sufficient for analysis [8]. Previous literature suggests that saturation is achieved at between 6 and 12 interviews [8,9]. We predetermined our sample sizes to ensure saturation in relevant subgroups (i.e., adopters and non-adopters) because it was not possible to transcribe and analyze the data as it was collected. Using a purposive sampling approach, we included all households in the study area who had agreed to a contract with the firm and a random sample of those who had declined a contract. Our final sample consisted of 48 interviews from 24 households– 16 adopter households and 8 non-adopter households—and was sufficient for saturation in all major themes.

Baseline stove ownership among the sample indicates that all adopter households own a charcoal stove (77% portable, 33% fixed). Nine percent of households have a traditional fuelwood stove and 9% have a Liquefied Petroleum Gas or elective stove. Among primary cooks, 78% are female, 85% are literate, and 35% are hired into the household.

We conducted two interviews within each household–one with the main decision maker and one with the main cook. In some households, the main decision maker and the main cook were the same person. In those cases, that person was asked to answer questions about decision making and cooking. Among adopters, 32 interviews were completed consisting of 13 female and 3 male decision makers as well as 12 female and 4 male main cooks. The decision maker and main cook were the same individual in 11 of the 16 adopter households. Non-adopters completed 16 interviews, including 8 females and 1 male. The decision maker and main cook were the same person in 7 of the 8 non-adopter households.

### Data collection

Two US-based investigators (VLF, PJ) trained the Rwandan team from Laterite, including five research assistants and one supervisor. During the three-day training, we discussed procedures for screening participants, ethical conduct of research, and interviewing techniques. We also reviewed the interview guides and consent forms in English and Kinyarwanda. All members of the data collection team had previous experience conducting qualitative interviews. University of North Carolina investigators developed the question guides in English. They were translated into Kinyarwanda by Laterite staff and pilot tested by the Rwandan team before starting data collection. The question guide for main decision-makers included sections on cooking, decision-making about cookstoves and fuels, experiences with the firm, and experiences with the stoves and fuels (adopters only). The question guide for the main cooks who were also main decision-makers included sections on cooking practices and experiences with the improved stoves and fuels (adopters only). The guide for main cooks who were not decision-makers focused on cooking practices; decision-making about cooking, stoves, and fuels; and experiences with the firm, and with the stoves and fuels (adopters only). Question guides in English and Kinyarwanda are included as supplementary materials.

The trained Rwandan research assistants conducted the interviews in the homes of the main decision-makers after obtaining written informed consent from all participants. The research assistants digitally recorded the interviews then transcribed them verbatim in Kinyarwanda and translated them into English. Laterite staff checked 10% of the transcripts for accuracy in transcription and translation.

### Data analysis

We analyzed the data using thematic content analysis, a form of descriptive analysis that allows us to understand and describe predominant factors affecting adoption and use of the pellet and ICS system in this setting, as reported by participants [10]. Author RS developed *a priori* deductive codes based on the interview guides and inductive codes based on emerging topics from the interviews. Following a review of all interviews, RS developed a codebook based on salient topics (Table 1) and subsequently coded the data using Dedoose (version 7.5.9), a qualitative analysis software program. Author VLF validated codes and code application periodically throughout analysis. Using the coded data, we developed salient themes based on deductive and inductive codes and created data matrices to facilitate further analysis and selection of illustrative quotations [11].

**Table 1:** Code book excerpt.

Code	Definition	Example Quote
Barrier to adoption	Factors that inhibit the adoption of the improved cooking system	“There is nothing that can make me decide to become their client as I have heard that their cookstoves burn food and cooking dishes, and as result the cooking dishes are perforated.”
Facilitator to adoption	Factors that promote the adoption of the improved cooking system	“I chose inyenyeri cookstoves because they are clean and they do not produce smoke.”
Factors considered in fuel type selection	Factors considered by cooks and decision makers in selecting fuel type	“The first thing I consider is the economy. The second is how long they require to cook and that they don’t make my utensils dirty.”
Factors considered in stove type selection	Factors considered by cooks and decision makers in selecting stove type	“Sometimes, wanting to prepare a meal quickly, I prefer the cookstove that will help me to be quick. Sometimes I also compare different types of cookstoves and choose the one which is not expensive or which doesn’t consume a high quantity of cooking fuel.”
Barrier to exclusive use	Factors that inhibit the exclusive use of the improved cooking system	“Sometimes we stopped because we didn’t have the fuel pellets, as most of the time the fuel pellets don’t last for a month. So in that case, we bought charcoal and used the charcoal stove instead.”
Barrier to fuel pellet use	Factors that inhibit the use of the fuel pellet component of the improved cooking system	“The only problem is that we are not allowed to buy fuel pellets anytime we want. Even when the fuel pellets have finished, we are supposed to wait for the purchasing date.
Barrier to stove use	Factors that inhibit the use of the stove component of the improved cooking system	“You have to charge them. If you could charge them for one or even two days and then use it for three or four days, that would be very helpful, not charge them every day because electricity is a problem”
Facilitator to fuel pellet use	Factors that promote the use of the fuel pellet component of the improved cooking system	“I am happy about the cleanliness of Inyenyeri fuel pellets. Charcoal used to make my hands dirty and I had to wash them after touching the charcoal. I used to have cuts all over my hands due to the charcoal. I do not get dirty whenever I use fuel pellets.”
Facilitator to stove use	Factors that promote the use of the stove component of the improved cooking system	“Mimi moto stoves work very well. You can use them anywhere, because they are very clean and have a nice shape, they don’t cause dirtiness.”
Cessation of use	Discontinued use of the improved cooking system by a household	“We stopped using it long time ago…because their cooking fuels don’t last long.”

## Results

### Facilitators to adoption and use of improved cooking energy system

Among adopters, key themes regarding facilitators to adoption and use of the improved system included: cooking time, cleanliness and low smoke production, cost savings, and peer encouragement.

#### Cooking time

Cooking time was the most frequently reported facilitator to use and the second most frequently mentioned facilitator to adoption. The rapid cooking feature of the stove is a key component of Inyenyeri advertising and multiple participants reported that marketing messages concerning reduced cooking times was an important factor in their decision to adopt. One participant described how the stove’s ability to cook quickly, as explained by a firm employee, played an important role in her decision to try the stove. She said:

*He [Inyenyeri employee] was saying that they are good stoves*, *easy to use and fast*, *and the food gets ready quickly*, *even beans don’t take as long as usual*, *and we were impressed by the fast acting product*. Female Decision Maker and Main Cook, Adopter

Following adoption of the stove, cooking time acted as a facilitator to use of the improved system. Many adopters reported that cooking foods with the improved system greatly reduced their cooking time when compared to traditional charcoal stoves. Several participants specified the amount of time saved when cooking certain dishes. For example, one participant reported the following:

*The time I used to spend preparing meals using charcoal has decreased*. *Before*, *it could take three hours to cook beans*, *while now I only use an hour* …*life is easier since I started using Inyenyeri cookstoves… I used to spend two hours preparing meals*, *but now I spend 30 minutes or one hour depending on the type of food I’m preparing*. Male Decision Maker and Main Cook, Adopter

These finding suggest that incorporating quick cooking time as a component of stove function and in marketing messages may bolster both adoption and use of ICS.

#### Cleanliness and low smoke production

Cleanliness was consistently discussed by adopters as a facilitator to both adoption and use. When describing the improved cleanliness associated with use of the improved cooking system, participants mentioned clean handling of the fuel pellets, as well as clean cooking pots and a clean home compared to their use of charcoal, the baseline fuel which is typically very dirty to handle.

Low smoke production was also an important feature among adopters. Several participants reported that the combination of low smoke production and overall cleanliness was key to their decision to adopt. One participant stated it quite simply when she said:

*I chose Inyenyeri cookstoves because they are clean and they do not produce smoke*. *You can even use them in the living room*. Female Decision Maker, Adopter

In addition to the stove’s limited smoke production, clean handling was a facilitator to use. One participant noted that fuel pellets are much cleaner to handle than charcoal. He said:

*One thing I am happy about is the cleanliness of Inyenyeri fuel pellets*. *The charcoal used to make my hands dirty and I had to wash them frequently after touching the charcoal*. *I used to have cuts all over my hands due to the charcoal*. *I do not get dirty when I use fuel pellets*. Male Main Cook, Adopter

Compared to charcoal stoves, the improved cooking system allowed users to store fuel and cook in different and often more convenient locations without concern for cleanliness or health issues. These benefits were reported by several adopters and are likely important drivers being this facilitator.

#### Cost savings

Cost savings was another frequently-mentioned factor promoting adoption and use. As a facilitator to adoption, several participants highlighted the price comparison between what they used to spend on charcoal and their current expenditure on fuel pellets. One participant said:

*I realized that it [Inyenyeri cookstove] might be beneficial to me because I used to use one sack of charcoal that cost 11*,*000 Rwandan francs*, *but with pellets I am able to use 45 kg per month that costs me 6*,*000 Rwandan francs*. Male Decision Maker and Main Cook, Adopter

Regarding use, adopters consistently mentioned their cost savings associated with the improved cooking system. Speaking to the benefits of using the system, one participant said:

*R*: *The advantage is the cost reduction*, *I used to buy charcoal for 11*,*000 Rwandan francs*, *but now pellets cost me 4*,*000 only*.

*I*: *That is enough for a month*?

*R*: *Yes*, *it is almost 30 kilos*. Female Decision Maker and Main Cook, Adopter

Quantitative findings among this population show that between 2012 and 2017 per kilogram Inyenyeri fuel pellet prices were on average 40% lower than the cost of charcoal and that exclusive Inyenyeri users require 15–20 fewer kilograms of fuel per month [7]. However, findings from this study as well as other qualitative studies [7,12,13], indicate that many adopters are not exclusive ICS users and use charcoal stoves and ICS concurrently. Therefore, although fuel pellet prices are lower and charcoal expenditures are reduced post-adoption, overall fuel expenditure remains relatively unchanged [7]. Although reduced cost was mentioned consistently as a facilitator to adoption and use, perceived cost savings, not actual cost savings, may be the actual facilitator in this case.

#### Peer encouragement

Specific to cookstove adoption, peer encouragement was an important facilitator as several adopter households reported that interaction with peers was a key step in their decision to adopt. While some were first introduced to the stove by peers, others reached out to peers after learning of the stove for validation of their decision to adopt.

For those adopters who were first introduced to the stove by peers, recommendations of the improved cooking system were often based on the aforementioned facilitators to adoption (e.g., cooking time, cleanliness, and cost). Positive peer interaction regarding the stove was an important factor in many participants’ decision to adopt. One participant noted that her discussions about the stove with a neighbor were centered on its time-saving and cost-reducing benefits. She said:

*R*: *I heard it from my neighbor who was using the Inyenyeri cookstove* …*She told me that it is better to use fuel pellets*, *in case you don’t have much time to prepare meals*. *She also told me that using the Inyenyeri cookstove helps with financial issues because it reduces the kitchen expenses*. Female Decision Maker, Adopter

For those who reached out to peers after being introduced to the stove by Inyenyeri promotions, peer encouragement was an important, mediating step in their decision to adopt. For example, one participant reported the following:

*When they [Inyenyeri staff] called me*, *I did not go there immediately*. *I went to ask a neighbor who has been using them [Inyenyeri cookstoves]*. *I told her that I received a call saying that I should go and get the Inyenyeri cookstoves*. *I asked her whether they were good and she told me that they were good*, *that I had delayed in getting them*. Female Decision Maker and Main Cook, Adopter

Peer interaction was an important factor prior to adoption, especially for those who were undecided. Positive peer feedback concerning the stoves transcended other major facilitators, suggesting that positive user experiences with various stove features may be a strong facilitator to adoption among peers and promote widespread dissemination of ICS.

### Barriers to adoption and use of the improved cooking energy system

Barriers to adoption were reported by non-adopters only, while barriers to use were discussed exclusively by adopters. We found significant overlap between these groups regarding barriers, such as damage to cooking pots, overcooked food, and cost. Peer discouragement was a significant barrier to adoption among non-adopters (contrary to the positive peer influence experienced among adopters) and purchasing protocol and excessive smoke production were major barriers to use among adopters.

#### Damage to cooking pots and overcooked food

Damage to cooking pots and overcooked food was reported as a barrier to both adoption and use by the majority of adopter and non-adopter households. This barrier was often associated with the need to constantly be near the stove, which was problematic because many participants reported that they were accustomed to performing other household chores or duties while preparing food using traditional charcoal stoves. One non-adopter explained that she cannot always be near the stove and, therefore, would not use the stove for fear of overcooking food. She said:

*Another issue is that those Inyenyeri cookstoves require you to be near while you are cooking*, *otherwise your food gets burnt*. *Since I am not always around the stove when I cook*, *I wouldn’t buy their stoves*. Female Decision Maker and Main Cook, Non-Adopter

For some non-adopters, this feature was the primary reason behind their decision to forego adoption of the stove. One participant noted that feedback from other users of the stove concerning its tendency to overcook food and damage cooking pots prompted him to forego adoption of the stove. He said:

*I was told that Inyenyeri cookstoves require constant follow up in order to prevent food from overcooking* …*Some women told me that they returned the Inyenyeri stoves because they damage their cooking pots and overcook food*. *They advised me not to use the Inyenyeri cookstoves since they are not good*. *That is why I do not use them*. Female Decision Maker, Non-Adopter

Although the stove is equipped with a fan adjustment feature which regulates heat production, one adopter reported that issues of burnt food and cooking pots persist regardless of the ability to adjust the stove’s heat level. He said:

*The cookstove is really good except that it burns food*. *It is necessary to remain near the stove and constantly check the food while cooking*, *otherwise it burns the food and the cooking pot as well*…. *I could reduce the heat but still*, *it destroys the cooking pots and burns the food… The point is*, *they [Inyenyeri stoves] overcook things a lot and that is why we usually use the other one [charcoal stove] in the kitchen*. Male Decision Maker and Main Cook, Adopter

However, other adopters offered contradictory views and explained that adjusting the heat and understanding how the stove functions diminishes the likelihood of overcooked food and damaged cooking pots. One adopter said:

*You could cook in the room using those stoves [Inyenyeri cookstove]*, *and in case you know very well their functioning*, *you would leave them and by the time you come back the food would be ready*, *you would just adjust the heat so that the food is not burnt*. Female Decision Maker and Main Cook, Adopter

Another adopter suggested that, beyond understanding how the stove functions, using quality cooking pots is an important factor in addressing this issue. She said:

*R: The problem is that some people do not know how to use them… The Inyenyeri staff trains people on how to use the cookstoves, but some people have poor understanding…*.

*I: I heard people saying that due to a lot of heat it releases*, *it causes damage to cooking pots. Has it happened to you?*

*R*: *Those are the ones who don’t know how Mimi Moto works*. *You cannot use bad cooking pots or fake ones*. *Look at my kitchen pots*, *they are really good*. *The cooking pots that are easily broken are the cheap ones*. *They need to buy original materials*. *Apart from buying good cooking pots*, *they also need to make sure that they regulate the heat of the cookstove*. *If you keep it on level 4 with your poor materials*, *you should expect them to get broken*. Female Decision Maker and Main Cook, Adopter

The high frequency of both non-adopters and adopters reporting issues related to damaged cooking pots and overcooked food, in conjunction with the contradictory views on the subject, suggest that this is an important barrier than may be mitigated through improved training related to correct stove use or including high-quality cooking pots as part of the improved cooking system.

#### Cost

Half of non-adopters and more than one-third of adopter households reported barriers related to cost. Significant barriers related to cost included start-up cost among non-adopters and fuel pellet price increases among adopters. Several non-adopters noted that the initial cost of the improved cooking system was too expensive and a barrier to adoption. As previously noted, start-up costs associated with the system include a one-time leasing fee of 5,000 Rwandan francs (approximately six US Dollars) and a monthly payment contract for fuel pellets, ranging from 4,000 to 8,000 Rwandan francs. Below, a non-adopter explains that she would have adopted the stove if she could have afforded it. She said:

*It is not because I didn’t like their products*. *As I told you*, *it is because I couldn’t afford it* …*I was already using the charcoal*, *when Inyenyeri brought theirs here*. *So*, *I couldn’t afford to buy another cookstove while I already had one* …*otherwise I would have bought it*. Female Decision Maker and Main Cook, Non-Adopter

Rising costs of fuel pellets was noted as a barrier among adopters. Pellet prices increased from 125 to 200 Rwandan francs per kilogram in 2016. One participant who had stopped using the stove stated that the price increase negated the pellets’ cost benefits over charcoal. She said:

*I didn’t mind using them [Inyenyeri fuel pellets]*, *however*, *they made it impossible for us to keep using them… They have increased pellets prices from 125 Rwandan francs per kilo to 200 per kilo*, *and at that price you actually spend more than what you would spend on charcoal*. *Everything else was fine except the dramatic price increase*.—Female Decision Maker, Adopter

Many participants were accustomed to purchasing small amounts of charcoal periodically throughout the month, suggesting that larger amounts of money were not readily available for cooking-related purchases. Although multiple participants reported cost as a facilitator to adoption, the initial lump sum required at startup was too much for some to afford. Similar findings regarding upfront investment costs have been previously reported [14]. Moreover, increases in the cost of fuel pellets proved to be a barrier to use for some adopters who may have considered cost a facilitator upon adoption.

#### Peer discouragement

Among non-adopters, peer discouragement was reported by six of the eight households as a barrier to adoption. Each time peer discouragement was mentioned, it was concerning the tendency of the ICS to damage cooking pots and overcook food. For example, one non-adopter reported that her decision not to become an Inyenyeri client was based on negative feedback from her friend concerning damage caused by the stove. She said:

*R*: *I wanted to buy it [Inyenyeri stove]*, *but I heard that you must always be near—if you want to cook you put everything near you*. *Otherwise*, *when you go far*, *it creates holes in the cooking pots*.

*I*: *And from whom did you first hear that information*?

*R*: *I heard it from people who use them* … *It is my friend who told me that* …*after that negative testimony*, *I decided not to become their client*. Female Decision Maker and Main Cook, Non-Adopter

Peer discouragement was a significant factor in non-adopters’ decision to forego adoption of the improved cooking system and was one of the two most frequently-reported barriers to adoption. Although peer interaction played a significant role in discouraging adoption, it was only mentioned in conjunction with a single barrier—damaged cooking pots and overcooked food. As such, these findings offer an important insight regarding training or redesign priorities for the firm.

#### Purchasing protocol

Among adopters, the protocol for purchasing fuel pellets was reported as a barrier to use by almost all adopter households. When households adopt the improved cooking system, they sign a contract to purchase a specified amount of fuel pellets at a set price on a certain day each month. The price each household pays for fuel pellets is dependent on the quantity of pellets, which is dependent on the number of stoves leased by a given household. For example, a household leasing a single stove would be assigned to purchase 30 kg of pellets at 4,000 Rwandan francs while households leasing two or three stoves would purchase 45 kg or 60 kg of pellets at 6,000 or 8,000 worth of pellets, respectively. If additional fuel pellets are needed prior to the designated purchasing date, pellets are sold per kilogram at a more expensive price and households are still required to purchase their full monthly pellet allotment on their next scheduled purchasing date.

Some participants may not have understood the option to purchase additional pellets at a per kilogram price as several reported that purchasing protocol was problematic due to the inability to buy fuel pellets before their purchasing date, even if they had run out of pellets. One participant pointed out that, because of this protocol, when she runs out of fuel pellets she is compelled to use other types of cooking fuel while awaiting her purchasing date. She said:

*R: They [Inyenyeri] gave us the purchasing date. For instance, I have to buy the fuel pellets on the 20th, otherwise, they could not allow me to buy before then*.

*I: What if your fuel pellets are finished on the 17th*, *wouldn’t you be allowed to buy?*

*R*: *No*, *I have to wait until my purchasing date… You have to find some other cooking fuels to cook with*. Female Decision Maker and Main Cook, Adopter

Others who were aware of the protocol for purchasing additional fuel pellets explained that the more expensive per-kilogram price was a barrier to use. One participant who had discontinued use of the stove reported the following:

*You have to buy pellets on your purchasing day*, *and if pellets are finished and you need some more*, *you need to buy per kilo* …*and that was more expensive than buying in bulk*. *So I realized it was not going to change and decided to stop using it [Inyenyeri stove]*. Female Decision Maker and Main Cook, Adopter

Several participants reported that the monthly supply of fuel pellets they were assigned to receive by staff (typically based on household size) consistently ran out before the month was over. This, in addition to the firm’s policy of not selling on credit, affected participants’ monthly budgets. Discussing the effects of running out of fuel pellets before the end of the month, one participant said:

*It leads us to misuse money by buying other cooking fuel*, *yet it was not planned in the budget… They [Inyenyeri] definitely can’t sell to us on credit*. *I once went there to tell them that my fuel pellets have finished*, *but I didn’t have money*. *They told me that I have to wait until I have the money*, *otherwise*, *they won’t sell to me on credit*. Female Decision Maker and Main Cook, Adopter

Potential misunderstandings regarding the option to purchase additional fuel pellets, higher per-kilogram prices, inaccurate monthly fuel supplies, and the inability to purchase on credit were all notable barriers to ICS use among adopters.

#### Excessive smoke production

Excessive smoke production was reported as a barrier to use by more than half of adopter households. Some participants reported that, in spite of Inyenyeri’s advertising messages concerning low smoke production, the stove produced a large quantity of smoke. One participant reported the following:

*The stove would start to release smoke… They [Inyenyeri promoters] said that it was healthy and clean to the point you can even put it here and cook*, *but we would be constantly blowing our nose if we tried* … *Our neighbors had them too*, *but told my daughter that they are too smoky… They had difficulties using them due to the smoke they release*. Female Decision Maker and Main Cook, Adopter

Another participant noted that the amount of smoke produced by the improved cookstove was even more than that produced by traditional charcoal stoves. He said:

*Sometimes fuel pellets cause too much black coating on our cooking pots because they produce a lot of smoke while charcoal didn’t produce a lot of smoke… With fuel pellets*, *cooking pots are coated with too much black soot*, *compared to when we used charcoal*. Male Decision Maker and Main Cook, Adopter

Although the majority of adopter households reported excessive smoke production as a barrier to use, nearly one-third of households reported low smoke production as a facilitator to use. In light of this discrepancy, one participant pointed out that the inconsistency could be related to operational issues of individual stoves, as some tend to produce smoke at the outset while others function well for some time before producing smoke. She said:

*The only problem is that sometimes they release smoke more than firewood does*, *and they form soot on cooking dishes*. *However*, *they [Inyenyeri staff] told us that it is a problem with the cookstove*. *It seems like their cookstoves are not made the same way*, *because sometimes you get a stove that releases smokes and when you approach them for a replacement you receive one that is functioning*, *but after some time it starts releasing smoke again*. Female Decision Maker, Adopter

Other participants offered varying explanations as to the cause of the smoke. One participant reported that using kerosene to ignite the pellets produced smoke while another reported that the use of kerosene mitigated smoke production. One participants noted that smoke production only occurs upon ignition and another suggested that smoke is only produced when the pellets are extinguished. In any case, reported levels of smoke production were contradictory and the mixed explanations regarding its cause suggest that variable knowledge concerning the correct use and function of the stove may be the primary cause of the inconsistencies.

## Discussion

Our findings suggest that factors affecting adoption and use of ICS are complex and sometimes contradictory. Peer influence was both a major barrier and an important facilitator to adoption. Reports of smoke production were also conflicting, noted as both a facilitator and barrier, and contrasting remarks regarding damage to cooking pots and overcooked food added to the complex nature of our findings.

### Peer influence

We found that influence from peers (e.g., friends and neighbors) outside of the household transcended nearly all other major facilitating and impeding factors related to adoption of the improved cooking system. For adopters, positive peer influence focused mainly on reduced expenses and cooking time and increased cleanliness, representing three of the four major facilitators. For non-adopters, negative peer feedback centered on damage to cooking pots and overcooked food, the two most-frequently reported barriers to adoption. Both adopters and non-adopters noted that peer interactions were a mediating step between contact with Inyenyeri employees and their decision to adopt the stove, suggesting that interactions with peers are a stronger determinant of adoption or non-adoption than interactions with ICS promoters.

As the mediating effect of peer influence spanned both adopters and non-adopters and acted as both a barrier and facilitator to adoption, we found this to be a key factor that may be significant to other ICS programs. Although related research has suggested the importance of assessing social factors in the dissemination of ICS [15], most studies have focused on social influences extraneous to peer interactions. Some researchers have focused on the social dynamics surrounding normative gender roles within patriarchal communities (e.g., decision making, cooking responsibilities, etc.) and how these cultural practices affect women, who are the primary users of cookstoves [4,6,16]. Others have assessed the societal effects of replacing traditional, deep-rooted cooking practices with ICS [6,17], while still others have explored the social and cultural effects of factors such as cooking time and cost, but did not discuss how social interactions influence these factors [18]. Overall, research addressing the effects of peer influence on adoption of ICS is limited and the subject remains largely unexplored [19].

Contrary to the idea that peer influence is a minor factor and does not play a significant role in the dissemination of ICS [4], we found it to be an important factor in implementation in Rwanda, especially among non-adopters. Other research suggests that positive peer interactions lead to favorable opinions of ICS, but do not improve actual adoption [20]. Although some adopters in our study reported peer influence to be an important factor related to adoption, nearly all non-adopters reported peer interactions to be a significant reason for their choosing to forego adoption. Therefore, while peer influence in general was a factor among adopters and non-adopters in our study, negative peer influence may be most important in relation to ICS non-adoption. These findings are in line with others demonstrating that social interactions play a more significant role in negatively affecting adoption than they do in positively supporting it [21].

These findings align with the Diffusion of Innovation theory, which underlines the importance of social interactions in the adoption of new technologies [22]. This theory categorizes the innovation decision process into five stages, the second of which, persuasion, relies on interpersonal connections and involves potential adopters actively seeking out information concerning the innovation. The theory suggests that adopters of an innovation reach out to earlier adopters before embracing the innovation and that peer communication is more effective in persuading individuals to adopt than other communication channels [22]. These points coincide with our findings and underline the significant effects of peer interaction across other barriers and facilitators to adoption.

### Adequate training and information

In addition to the effects of social interactions, barriers related to the adoption of a new technology had significant effects on adoption and use of the improved cooking system. New technology has been addressed as a barrier to the use of ICS in numerous studies [5]. Specific components of adoption and use of new technologies addressed by such studies include: motivation, affordability, required engagement, and benefits related to the use of new products, as well as stove design and its compatibility with cooking practices [4,23,24]. The findings from our study, which indicate that potential user error and related outcomes are important barriers to the adoption of new technologies, have not previously been reported.

Concerning barriers described by both adopters and non-adopters, damage to cooking pots, overcooked food, and excessive smoke were among the most significant. Although many adopter and non-adopter households reported that the ICS damaged cooking pots and overcooked food, other participants pointed out that these effects were the result of improper use of the stove. Likewise, reports of excessive smoke by adopter households were countered by a similar argument that misuse was the culprit.

Although incorrect use of the ICS may not be the sole cause of damage to cooking pots, overcooked food, and excessive smoke, it is plausible that improper use of the stove may be an important factor underlying these barriers to adoption and use. Previous findings indicate that insufficient user training is a considerable barrier to adoption of ICS [25]. The results of our study support these findings and suggest that comprehensive trainings and instruction regarding the correct use of new technologies are important factors in dissemination. As such, employing innovative, context-appropriate communication mediums such as posters, text messages, or radio advertisements to inform users regarding stove operations and their correct use may help to address these issues and bolster adoption and use of innovative, ICS.

Finally, an interesting dimension of conducting research on an intervention led by a for-profit social venture is that they are continually learning and trying to improve their marketing and customer support services. By design, the business model requires that they continually update and innovate. Our study was conducted relatively soon after Inyenyeri began to scale-up after the initial pilot phase of their business. Many of the issues elaborated in our analysis as barriers to adoption are well known to the firm’s customer service representatives. To the extent possible, they are working to mitigate them.

## Limitations

This study has some potential limitations. It is possible that salience bias contributed to the numerous reports of peer interactions (especially negative), as these are likely conversations easily recollected by participants. In addition, courtesy bias may have led to inaccurate positive feedback regarding the improved cooking system by participants providing information they thought the interviewer wanted to hear. If present, this bias may explain why certain stove features were reported as both facilitators and barriers to adoption.

## Conclusions

Understanding barriers and facilitators to the adoption and use of sustainably produced fuel pellets and ICS, especially in countries that rely largely on solid fuels and traditional cookstoves, such as Rwanda, has major implications for global public health. In addition to supporting other findings concerning more commonly understood barriers and facilitators to ICS adoption and use, such as cost, cooking time, and cleanliness, our findings suggest that peer influence and adequate training and information play crucial roles in the spread of ICS. Peer influence played a significant role as both a facilitator and a barrier to adoption. Peer feedback from adopters within the community transcended other key barriers and facilitators and was found to be an important step for many participants in their decision to adopt. Additionally, mixed feedback concerning smoke production, cooking pot damage, and overcooked food suggests that limited training and information regarding the correct use of the improved cooking system was the primary cause of these barriers to adoption and use. We suggest that future ICS programs prioritize communication to peer networks and provision of adequate training on ICS systems to improve rates of adoption and use and, ultimately, improve the health of communities.

## Supporting information

S1 File Interview Guides. (ZIP)
