# Supplementary material for: Barriers and facilitators to adoption and use of fuel pellets and improved cookstoves in urban Rwanda
Source: PLoS One. 2018 Oct 8;13(10):e0203775. doi: 10.1371/journal.pone.0203775 (PMC6175269; doi:10.1371/journal.pone.0203775)
Supplement: S1 File — (ZIP) [file pone.0203775.s001.zip › Interview Guide for Cooks Who are NOT Decision Makers (English & Kinyarwanda).docx]

**The Health and Poverty Effects of a Household Energy Initiative in Rwanda**

**Uruhare rw’umushinga w’amashyiga atangiza ibidukikije (Household EnergyInitiative) ku buzima n’ubukene mu Rwanda**

**Qualitative In-Depth Interview Guide— MAIN COOKS WHO ARE NOT DECISION MAKERS**

**Inyoborabiganiro igamije gukusanya ibitekerezo—Umuntu ushinzwe guteka**

Thank you very much for participating in this interview today. I would like to have a conversation with you to talk about your cooking practices, stove, and fuel preferences, and any experiences you have had with Inyenyeri. This is an informal conversation and there are no right or wrong answers. I am interested in anything that you want to share with me to help me learn more about the topics we are discussing.

Urakoze cyane kwemera kugira uruhare muri ubu bushakashatsi. Ndashaka ko tuganira ku uburyo uteka, uburyo uhitamo amashyiga n’ibicanwa n’uburyo washimye ibikorwa bya sosiyete Inyenyeri. Iki ni ikiganiro gisanzwe nta bisubizo byiza nta n’ibisubizo bifutamye. Ngutezeho ibisobanuro uri bumpe biza kumfasha gusobanukirwa kurushaho ibyo turi buganireho.

| **FOR ALL COOKS** | |
| --- | --- |
| **Cooking on a typical Day Umunsi wihariye** | |
| 1.1 | Tell me about cooking on a typical day. Mbwira ibyo guteka ku munsi wihariye |
| 1.2 | When do you cook? Ni ayahe masaha mutekeraho? |
| 1.3 | Who usually cooks with you? Ninde ugufasha guteka? |
| 1.4 | Where do you cook? Does cooking location vary? Ni hehe mutekera? Ese aho gutekera harahinduka?  *(Probe on why it varies, such as time of day, season, type of stove, type of fuel). (Gerageza kumenya impamvu aho gutekera hahinduka bitewe n’igihe runaka ku munsi, ikirere, ubwoko bw’amashyiga, ubwoko bw’ibicanwa bwakoreshejwe)* |
| 1.5 | What stoves do you use? Can you show me? Ni ayahe mashyiga mukoresha? Ese mushobora kuyanyereka?  ***[Make a list of ALL stoves they use.] [Erekana urutonde rw’amashyiga yose bakoreshwa]*** |
| 1.6 | What fuels do you use? Can you show me? Ni ubuhe bwoko bw’ibicanwa mukoresha? Ese ushobora kunyereka?  ***[Make a list of ALL fuels they may use.] [Erekana urutonde rw’ibicanwa bashobora gukoresha.]*** |
| 1.7 | How frequently do you use each stove? Ni kangahe ukoresha buri shyiga? |
| 1.8 | How frequently do you use each fuel? Ni kangahe ukoresha buri gicanwa? |
| 1.9 | How do you decide which stove to use? Ni gute uhitamo ishyiga ukoresha? |
| 1.10 | What are your favorite things to cook with each type of stove? Why? Ni ibiki ukunze guteka kuri buri bwoko bw’ishyiga? Tubwire impamvu kuri buri gisubizo watanze? |
| 1.11 | How do you decide which fuels to use when preparing a meal? Ni gute uhitamo igicanwa iyo ugiye guteka?  *(Probe on speed of cooking, cost, availability, health/ safety, environmental, social acceptability)* *Gerageza kubaza ibibazo bijyanye no gushya vuba, ibiciro, ukuboneka kwabyo, ubuzima/umutekano, kubungabunga ibidukikije, kuba ishyigikirwa na benshi)* |
| 1.12 | What fire starting materials do you use? Ni ibiki mukoresha mu gufatisha umuriro? |
|  |  |
| **Decision Making on Cooking - Main Cook Gufata icyemezo kerekeranye no guteka – Umuntu ufite uruhare runini mu guteka kurusha abandi** | |
| 2.1 | Who makes the decisions in your household about what types of food are cooked? Ninde ufata icyemezo cyerekeranye n’ubwoko bw’ibiribwa bigomba gutekwa mu muryango?  -Anyone else? –Nta wundi?  *(Probe: If more than one person, probe on their individual roles in decision making.)* (*Gerageza kumenya niba hari abantu barenze umwe, gerageza kumenya uruhare rwa buri wese mu gufata icyemezo)* |
| 2.2 | Who makes decisions in your household on which fuels are used for cooking? Ninde ufata icyemezo cyerekeranye n’ubwoko bw’ibicanwa bukoreshwa muri uru rugo?  -Anyone else? –Nta wundi?  *(Probe: If more than one person, probe on their individual roles in decision making.)* (*Gerageza kumenya niba hari abantu barenze umwe, gerageza kumenya uruhare rwa buri wese mu gufata icyemezo)* |
| 2.3 | With whom in your household do you talk to about types of fuels for cooking?  Ninde wo muri uru rugo uganira nawe ibijyanye no gufata ibyemezo by’ubwoko bw’ibicanwa bukoreshwa muri uru rugo? |
| 2.4 | With whom outside of your household do you talk to about types of fuels for cooking?  Ninde muntu wundi utari uwo muri uru rugo uganira nawe ibijyanye no gufata ibyemezo by’ubwoko bw’ibicanwa bukoreshwa muri uru rugo?  *(Probe on friends, relatives, clubs, associations, religious, community)*  *(Gerageza kumenya niba hari incuti, abavandimwe, ama calabu, amatsinda , abihayimana,cyangwa abantu baho atuye)* |
| 2.5 | Who makes decisions in your household on which stoves are used for cooking? Ninde ufata icyemezo ku bijyanye n’ubwoko bw’amashyiga bukoreshwa muri uru rugo?  -Anyone else? –Nta wundi?  *(Probe: If more than one person, probe on their individual roles in decision making.)* (*Gerageza kumenya niba hari abantu barenze umwe, gerageza kumenya uruhare rwa buri wese mu gufata icyemezo)* |
| 2.6 | With whom in your household do you talk to about types of stoves for cooking?  Ninde wo muri uru rugo uganira nawe ibijyanye no gufata ibyemezo by’ubwoko bw’amashyiga akoreshwa muri uru rugo? |
| 2.7 | With whom outside of your household do you talk to about types of stoves for cooking?  Ninde muntu wundi utari uwo muri uru rugo uganira nawe ibijyanye no gufata ibyemezo by’ubwoko bw’amashyiga akoreshwa muri uru rugo?  *(Probe on friends, relatives, clubs, associations, religious, community) (Gerageza kumenya niba hari incuti, abavandimwe, ama Kalabu, amatsinda , abihayimana,cyangwa abantu baho atuye)* |
|  |  |
| **Experiences with Inyenyeri Kumenya sosiyete Inyenyeri** | |
|  |  |
| 3.1 | What do you remember hearing about the reasons why you should use the Mimi moto or Philips stove? Ni ibiki wibuka igihe wumvise bwa mbere impamvu ugomba gukoresha amashyiga yakozwe |
| 3.2 | Who did you hear this from? Ibi wabyumviye kuri nde? |
| 3.3 | Do you know of the company Inyenyeri? Ese waba uzi sosiyete yitwa Inyenyeri? |
| **IF NO AND HOUSEHOLD IS AN INYENYERI CUSTOMER, GO TO SECTION 4 OF THE INTERVIEW GUIDE**  **IF NO AND HOUSEHOLD IS NOT AN INYENYERI CUSTOMER, GO TO SECTION 6 OF THE INTERVIEW GUIDE**  **IF YES, CONTINUE TO THE NEXT QUESTION (3.4).** | |
| 3.4 | How did you first learn about Inyenyeri? (Niba ari yego) Ni gute wamenye sosiyete Inyenyeri bwa mbere? |
| 3.5 | Tell me about your experiences with Inyenyeri. Tubwire uko ubona ibintu byakozwe na Inyenyeri. |
| 3.6 | How does Inyenyeri interact with you as a cook? Ni gute Inyenyeri ikorana nawe nk’umuntu uteka? |
| 3.7 | What is positive about your interactions with Inyenyeri? Ni iki ushima mu mikoranire yawe na sosiyete Inyenyeri? |
| 3.8 | What is challenging about your interactions with Inyenyeri? Ni izihe mbogamizi uhura nazo mu mikoranire yawe na sosiyete Inyenyeri? |
| 3.9 | How often have you met with Inyenyeri Customer Service Representatives? Wahuye kangahe n’abakozi bashinzwe kumenyekanisha ibikorwa bya sosiyete Inyenyeri? |
| 3.10 | For what reasons? Kubera iyihe mpamvu? |
|  |  |
| **FOR HOUSEHOLDS THAT ARE INYENYERI CUSTOMERS ONLY IBIBAZO BYAGENEWE ABAKIRIYA BA SOSIYETE INYENYERI GUSA** | |
| **Experiences with Phillips stove and biomass fuel pellets.** **Amashyiga ya phillips n’ibicanwa bikozwe mu bishingwe bya sosiyete Inyenyeri** | |
| I’d like to ask you about using the Phillips stove and Biomass Pellets Ndifuza kukubaza ibibazo byerekeranye no gukoresha amashyiga ya Phillips ndetse n’amakara akozwe mu bishingwe | |
| 4.1 | How long did you use a Phillips stove? Ese amashyiga ya phillips mwayakoreshseje igihe kingana iki ? |
| 4.2a | How many Phillips stoves did you have? Ese mwari mufite amashyiga ya Phillips angahe? |
| 4.2b | (If the household had more than one Phillips stove), How do you use them at the same time? (Niba umuryango warufite amashyiga ya phillips arenze rimwe) ni gute yakoreshwaga icyarimwe? |
| 4.3 | How did your use of the Phillips stove change over time, why?  Ni gute ikoreshwa ry’amashyiga ya phillips muri uru rugo ryagiye rihinduka uko igihe cyagiye gitambuka ? Mwatubwira impamvu yabyo? |
| 4.4 | Did you ever stop using the Phillips stove for a period of time? Tell me about this. Ese mwaba mwarigeze mureka gukoresha amashyiga ya phillips mu gihe runaka? Gira icyo ubitubwiraho. |
| 4.5 | When using the Phillips stove, which fuels did you use? [*Name all fuels].* Ni ibihe bicanwa mwakoreshaga mu mashyiga ya Phillips? [*Vuga amazina y’ibyo bicanwa byose].* |
| 4.6 | What did you like about the Phillips stove compared to other stoves?  Ni ibiki mwaukundiraga amashyiga ya Phillips ugereranije nayandi mashyiga? |
| 4.7 | What did you dislike about the Phillips stove compared to other stoves?  Ni ibiki mwagayaga amashyiga ya Phillips ugereranije nayandi mashyiga? |
|  |  |
| **Experiences with Mimi Moto stove and biomass fuel pellets.** | |
| Now I’d like to ask you about using the Mimi moto stove and Biomass Pellets Ubu noneho ndifuza kukubaza ibibazo byerekeranye no gukoresha amashyiga ya Mimi moto ndetse n’amakara akozwe mu bishingwe | |
| 5.1 | How long have you been using a Mimi Moto stove? Umaze igihe kingana iki ukoresha amashyiga ya Mimi Moto ? |
| 5.2a | How many Mimi Moto stoves do you have? Ese ufite amashyiga ya Mimi Moto angahe? |
| 5.2b | (If the household has more than one Mimi Moto stove), How do you use them at the same time? (Niba umuryango ufite amashyiga ya Mimi Moto arenze imwe) ni gute akoreshwa icyarimwe? |
| 5.3 | Would you say you use it more often, less often, or the same as when you first got it? Ese ushobora kwemeza ko murikoresha kenshi, gake cyangwa kimwe n’igihe mwariguraga? |
| 5.4 | If it has changed over time, why? Niba byarahindutse uko igihe cyagiye gitambuka, watubwira impamvu yabyo? |
| 5.5 | Have you ever stopped using the Mimi Moto stove for a period of time? Tell me about this. Ese waba warigeze ureka gukoresha amashyiga ya Mimi Moto mu gihe runaka? Gira icyo ubitubwiraho. |
| 5.6 | When using the Mimi Moto stove, which fuels do you use? [*Name all fuels].* Ni ibihe bicanwa mukoresha iyo mwifashisha amashyiga ya Mimi Moto? [*Vuga amazina y’ibyo bicanwa byose].* |
| 5.7 | What foods do you prepare using the biomass pellets? What do you like about the pellets compared to other fuels? Ni ibihe biribwa uteka ukoresheje amakara akozwe mu bishingwe? Amakara akozwe mu bishingwe muyakundira iki ugereranije n‘ibindi bicanwa? |
| 5.8 | Which foods do you prefer to cook using another fuel other than biomass pellets? Why? Ni ibihe biribwa ukunda guteka ukoresheje ikindi gicanwa kitari amakara akozwe mu bishingwe? Tubwire impamvu yabyo |
| 5.9 | What do you like about the Mimi Moto stove compared to other stoves?  Ni ibiki ukundira amashyiga ya Mimi Moto ugereranije nayandi mashyiga? |
| 5.10 | What do you dislike about the Mimi Moto stove compared to other stoves?  Ni ibiki ugaya amashyiga ya Mimi Moto ugereranije nayandi mashyiga? |
| 5.11 | How has use of Inyenyeri fuel and stoves changed the amount of time you spend cooking?  Ni gute ikoreshwa ry’amashyiga n’ibicanwa byakozwe n’Inyenyeri byahinduye amasaha/igihe mwakoreshaga mu mirimo yo guteka? |
| 5.12 | (If cooking with Inyenyeri fuel and stoves takes less time than before they had the Inyenyeri stove), how do you use your extra time?  Niba amasaha wakoreshaga mu mirimo yo guteka yaragabanutse kubera gukoresha amashyiga ndetse n’ibicanwa bya sosiyete inyenyeri, ese andi masaha asaguka uyamara ukora iki? |
| 5.13 | Would you recommend Inyenyeri fuel pellets to anyone you know? Why/why not? Ese washishikariza umuntu uzi kuba yakoresha amakara akorwa na Sosiyete Inyenyeri? Tubwire impamvu. |
| 5.14 | Would you recommend Inyenyeri cookstoves to anyone you know? Why/why not?  Ese washishikariza umuntu uzi kuba yakoresha amashyiga akorwa na sosiyete Inyenyeri?Yego/Oya? Watubwira impamvu? |
|  |  |
| **FOR ALL COOKS** | |
| **Wrap up and closing Kwibukiranya ibyaganiriweho no gusoza** | |
| 6.1 | Is there anything else you would like to add? Ese hari ikindi wifuza kongeraho ku byo twaganiriye? |
| 6.2 | Do you have any questions for me? Ese hari ikintu wifuza kumbaza? |

Thank you for talking with us today. Urakoze kutuganiriza uyu munsi
