# Supplementary material for: Barriers and facilitators to adoption and use of fuel pellets and improved cookstoves in urban Rwanda
Source: PLoS One. 2018 Oct 8;13(10):e0203775. doi: 10.1371/journal.pone.0203775 (PMC6175269; doi:10.1371/journal.pone.0203775)
Supplement: S1 File — (ZIP) [file pone.0203775.s001.zip › Interview Guide for Decision Makers (English & Kinyarwanda).doc]

**The Health and Poverty Effects of a Household Energy Initiative in Rwanda**

**Uruhare rw’umushinga w’amashyiga atangiza ibidukikije (Household Energy Initiative) ku buzima n’ubukene mu Rwanda**

**Qualitative In-Depth Interview Guide-- Main Decision Maker**

**Inyoborabiganiro igamije gukusanya ibitekerezo—Umukuru w’Umuryango/Umuntu w’ingenzi mu gufata ibyemezo**

Thank you very much for participating in this interview today. I would like to have a conversation with you to talk about groups in your community, your household’s cooking practices, stove and fuel preferences, and your experiences with the company, Inyenyeri. This is an informal conversation and there are no right or wrong answers. I am interested in anything that you want to share with me to help me learn more about you and your family.

Urakoze cyane kwemera kugira uruhare muri ubu bushakashatsi. Ndashaka ko tuganira ku buzima bw’abantu batandukanye mu mudugudu utuyemo, uburyo muteka, amashyiga n’ibyo mukunze gukoresha mu gucana ndetse n’uburyo mukorana na sosiyete Inyenyeri. Iki ni ikiganiro gisanzwe nta bisubizo byiza nta n’ibisubizo bifutamye. Ngutezeho ibisobanuro uri bumpe biza kumfasha kukumenya wowe ubwawe n’umuryango wawe.

| **FOR ALL HOUSEHOLDS IBIBAZO BIGENEWE IMIRYANGO YOSE** | |
| --- | --- |
| **Household Cooking Guteka mu muryango** | |
| 1.1a | In your household, who takes part in cooking food for the family? Mu muryango wawe, ninde ugira uruhare mu guteka?  (*Probe on any hired help, even if not full time, and on children who prepare any food.) (Gerageza kumenya niba hari umuntu wishyurwa kugira ngo atekere urugo, n’ubwo byaba ari iby’igihe gito, gerageza kumenya niba hari abana bateka)* |
|  | ***[List* *the name and relationship to the respondent of each person who helps with cooking. For each person, ask the following.]***  ***[Tubwire amazina n’isano ufitanye na buri muntu uteka. Kuri buri muntu, baza ibibazo bikurikira.]*** |
| 1.1b | How does this person help with cooking food? Ni gute uyu muntu afasha guteka ibiryo? |
| 1**.**1c | What other roles does [NAME] play in this household? Ni izihe nshingano zindi [VUGA IZINA] afite muri uyu muryango?  (*Probe on* *any other tasks, chores, work they responsible for in the family.)* (*Gerageza kumenya niba hari indi mirimo bakora mu muryango)* |
| 1.2 | Who does **most** of the cooking in your household? Ninde ugira uruhare runini mu gutekera umuryango? |
| 1.3 | Who makes the decisions in your household about what types of food are cooked? Ninde ufata icyemezo cyerekeranye n’ubwoko bw’ibiribwa bigomba gutekwa mu muryango?  -Anyone else? –Nta wundi?  *(Probe: If more than one person, probe on their individual roles in decision making.)* (*Gerageza kumenya niba hari abantu barenze umwe, gerageza kumenya uruhare rwa buri wese mu gufata icyemezo)* |
| 1.4 | Who makes decisions in your household on which fuels are used for cooking? Ninde ufata icyemezo cyerekeranye n’ubwoko bw’ibicanwa bukoreshwa muri uru rugo?  -Anyone else? –Nta wundi?  *(Probe: If more than one person, probe on their individual roles in decision making.)* (*Gerageza kumenya niba hari abantu barenze umwe, gerageza kumenya uruhare rwa buri wese mu gufata icyemezo)* |
| 1.5 | Who makes decisions in your household on which stoves are used for cooking? Ninde ufata icyemezo cyerekeranye n’ubwoko bw’amashyiga akoreshwa muri uru rugo?  -Anyone else? –Nta wundi?  *(Probe: If more than one person, probe on their individual roles in decision making.)* (*Gerageza kumenya niba hari abantu barenze umwe, gerageza kumenya uruhare rwa buri wese mu gufata icyemezo)* |
| 1.6 | Which cooking fuels do you use in your household? Any other types?  Ni ibihe bicanwa mukoresha mu rugo rwanyu? Ese hari ibindi mukoresha? |
| 1.7 | What factors do you consider when deciding what kind of fuel to use in your household? Ni ibiki ushingiraho mu guhitamo ubwoko bw’ibicanwa mu rugo rwanyu?  *(Probe: types of foods, cost, availability, health/ safety, environmental, social acceptability)* (*Gerageza kumenya niba biterwa n’ubwoko bw’ibiryo, ibiciro, kuba biboneka, ubuzima/umutekano, ibidukikije, kuba ishyigikirwa na benshi)* |
| 1.8 | With whom in your household do you discuss decisions about types of fuels for cooking?  Ninde wo muri uru rugo uganira nawe ibijyanye no gufata ibyemezo by’ubwoko bw’ibicanwa bukoreshwa muri uru rugo? |
| 1.9 | With whom outside of your household do you discuss decisions about types of fuels for cooking?  Ninde muntu wundi utari uwo muri uru rugo uganira nawe ibijyanye no gufata ibyemezo by’ubwoko bw’ibicanwa bikoreshwa muri uru rugo?  *(Probe on friends, relatives, clubs, associations, religious, community)*  *(Gerageza kumenya niba hari incuti, abavandimwe, ama calabu, amatsinda , abihayimana,cyangwa abantu baho atuye)* |
| 1.10 | What specifically do you talk about? Mu by’ukuri tubwire icyo muganiraho?  (*Probe:* *ask for specifics on each person mentioned.*) *(Gerageza kubaza ibibazo byihariye kuri buri muntu wavuzwe mu gisubizo)* |
| 1.11 | Are you generally satisfied with the cooking fuels you currently use? Tell me about this. Muri rusange, ese unyuzwe n’ubwoko bw’ibicanwa ukoresha ubu? Gira icyo utubwira kuri ibi. |
| 1.12 | Which cooking stoves do you use in your household? Any other types of stoves? Ni ubuhe bwoko bw’amashyiga mukoresha mu muryango? Ese hari ubundi bwoko bw’amashyiga mukoresha?  *(Probe for specific types of cookstoves, e.g., Philips, Mimi Moto, 3-stone, charcoal, etc.)* (*Gerageza kumenya niba bakoresha ubwoko runaka bw’amashyiga, urugero Philips, Mimi Moto, amashyiga agizwe n’amabuye atatu, amakara n’ibindi)* |
| 1.13 | What factors do you consider when deciding the type of stoves to use in your household? Ni iki ushingiraho ujya guhitamo ubwoko bw’amashyiga ugiye gukoresha mu muryango?  *(Probe: types of foods, cost, availability, health/ safety, environmental, social acceptability)* (*Gerageza kumenya niba biterwa n’ubwoko bw’ibiryo, ibiciro, kuba biboneka, ubuzima/umutekano, ibidukikije, kuba ishyigikirwa na benshi)* |
| 1.14 | With whom in your household do you discuss decisions about types of stoves for cooking?  Ninde wo muri uru rugo uganira nawe ibijyanye no gufata ibyemezo by’ubwoko bw’amashyiga akoreshwa muri uru rugo? |
| 1.15 | With whom outside of your household do you discuss decisions about types of stoves for cooking?  Ninde muntu wundi utari uwo muri uru rugo uganira nawe ibijyanye no gufata ibyemezo by’ubwoko bw’amashyiga akoreshwa muri uru rugo?  *(Probe on friends, relatives, clubs, associations, religious, community)*  *(Gerageza kumenya niba hari incuti, abavandimwe, ama calabu, amatsinda , abihayimana,cyangwa abantu baho atuye)* |
| 1.16 | What specifically do you talk about? Mu by’ukuri tubwire icyo muganiraho  (*Probe:* *ask for specifics on each person mentioned.*) *(Gerageza kubaza ibibazo byihariye kuri buri muntu wavuzwe mu gisubizo)* |
| 1.17 | Are you generally satisfied with the cooking stove you currently use? Tell me about this. Muri rusange, ese unyuzwe n’ishyiga ukoresha ubu? Gira icyo utubwira kuri ibi.  *(After they respond, probe on safety of stove and instances of burns for cook/ children.) (Nyuma yo gusubiza ikibazo, gerageza kumenya icyo batekereza ku mutekano ishyiga ritanga n’ingero z’uburyo itwika abateka cyangwa abana)* |
| 1.18 | What types of lighting materials do you use in your household? Any others?  Ni ibiki mukoresha mu gufatisha umuriro? Nta bindi?  *(Probe for specific types: plastic, kerosene, paper, grasses, small branches.)*  (*Gerageza kumenya niba bakoresha pulasitike cyangwa amashashi, peterori, ibyatsi, udushami duto tw’ibiti.)* |
|  |  |
| **FOR INYENYERI CUSTOMERS (ADOPTERS and SUSTAINED USERS): ABAKIRIYA BA INYENYERI (ABATANGIYE GUKORESHA IBIKORESHO BYA INYENYERI NDETSE N’ABASANZWE BAKORESHA IBIKORESHO BIKORWA NA INYENYERI)** | |
| **Experiences of adopters with Inyenyeri Imikoranire na sosiyete Inyenyeri** | |
|  |  |
| 2.1 | How did you first learn about Inyenyeri? Ni gute wamenye bwa mbere amakuru yerekeranye na Inyenyeri? |
| 2.2 | What do you remember hearing about the benefits of Inyenyeri’s biomass pellets and improved cookstove system? Tubwire icyo wibuka igihe wumvise ibyerekeranye n’akamaro k’amakara akozwe mu bishingwe n’amashyiga ya rondereza yakozwe na sosiyete Inyenyeri? |
| 2.3 | What do you remember hearing about why you should become an Inyenyeri customer? Tubwire icyo wibuka ku byo wabwiwe byerekeranye n’impamvu ugomba kuba umukiriya wa sosiyete Inyenyeri? |
| 2.4 | What do you remember about Inyenyeri’s marketing message (sales pitch)? Tubwire icyo wibuka ku butumwa bwo kwamamaza ibikorwa bya sosiyete Inyenyeri? (umubare w’ibikoresho bimaze kugurishwa)? |
| 2.5 | Why did you choose to become an Inyenyeri customer? Kuki wahisemo kuba umukiriya wa sosiyete Inyenyeri?  *(Probes: Inyenyeri marketing, customer service, health, costs, availability, environmental)* (*Gerageza kumenya niba biterwa n’ubwoko bw’ibiryo, ibiciro, kuba biboneka, ubuzima/umutekano, ibidukikije, kuba ishyigikirwa na benshi)* |
| 2.6 | Tell me about the process of becoming an Inyenyeri customer. Nsobanurira uburyo umuntu aba umukiriya wa sosiyete Inyenyeri. |
| 2.7 | How did Inyenyeri interact with you or other members of your household, or your cook before you became a customer? Ni gute sosiyete Inyenyeri yashyikiranaga nawe cyangwa undi muntu mu muryango wawe cyangwa umuntu ugutekera mbere yuko muyigana(Muba abakiliya)? |
| 2.8 | What was positive during this experience? Ni iki washimye muri uko gushyikirana? |
| 2.9 | What were any barriers to becoming an Inyenyeri customer? Ni izihe mbogamizi wahuye nazo zakubuzaga kuba umukiriya wa sosiyete Inyenyeri? |
| 2.10 | Tell me about your interactions with Inyenyeri customer service representatives since you became a customer. Kuva mwana abakiliya ba sosiyete Inyenyeri, tubwire uburyo mushyikirana n’abashinzwe kwakira abakiriya muri sosiyete Inyenyeri. |
| 2.11 | How often have you met with them? Mwahuye nabo kangahe? |
| 2.12 | For what reasons? Kubera iyihe mpamvu? |
| 2.13 | What are the positive aspects of being a customer? Ni izihe nyungu umuntu abona mu kuba umukiriya w’Inyenyeri? |
| 2.14 | What are some of the challenges associated with being an Inyenyeri customer? Ni izihe mbogamizi uhura nazo nk’ umukiriya wa sosiyete inyenyeri? |
| 2.15 | Have you ever stopped being an Inyenyeri customer for a period of time? Tell me about it. Waba warigeze uhagarika kuba umukiriya w’Inyenyeri mu gihe runaka? Gira icyo ubitubwiraho. |
| 2.16 | Would you recommend Inyenyeri fuel pellets to anyone you know? Why/why not? Ese washishikariza umuntu uzi kuba yakoresha amakara ya sosiyete Inyenyeri? Tubwire impamvu. |
| 2.17 | Would you recommend Inyenyeri cookstoves to anyone you know? Why/why not?  Ese washishikariza umuntu uzi kuba yakoresha amashyiga akorwa na Sosiyete Inyenyeri? Tubwire impamvu. |
| 2.18 | How does the Inyenyeri fuel and cookstove affect your costs related to cooking?  Tubwire ingaruka ibicanwa n’amashyiga bya sosiyete Inyenyeri byaba bigira ku mafaranga muba mwateganije gukoresha ku bijynaye n’amafunguro. |
|  |  |
| **FOR THOSE WHO CHOSE NOT BE BECOME INYENYERYI CUSTOMERS (NON-ADOPTERS): IBIBAZO BIGENEWE ABAHISEMO KUTABA ABAKIRIYA BA SOSIYETE INYENYERI (ABADAKORESHA IBYO SOSIYETE INYENYERI IKORA)** | |
| **Experiences of non-adopters with Inyenyeri , Ku bantu badakorana na Sosiyete Inyenyeri** | |
| 3.1 | Tell me about your experiences with Inyenyeri. Tubwire ibyerekeranye n’imikoranire yanyu na Inyenyeri. |
| 3.2 | How did you first learn about Inyenyeri? Ni gute wamenye bwa mbere amakuru yerekeranye n’ibikorwa bya sosiyete Inyenyeri? |
| 3.3 | What do you remember about Inyenyeri’s marketing message (sales pitch)? Tubwire icyo wibuka ku butumwa bwo kwamamaza ibikorwa bya sosiyete Inyenyeri? (umubare w’ibikoresho bimaze kugurishwa)? |
| 3.4 | Tell me about why you did not decide to be an Inyenyeri customer at this time. Tubwire impamvu utahisemo kuba umukiriya wa sosiyete Inyenyeri kugeza iki gihe?  *Probe on marketing, customer service representatives, word-of-mouth, costs, etc.)* (*Gerageza kumenya niba byatewe n’uburyo bamenyekanisha ibikorwa byabo, abakozi bashinzwe gutanga serivisi ku bakiriya, amagambo, ibiciro n’ibindi…)* |
| 3.5 | What might make you change your mind about becoming an Inyenyeri customer in the future? Ni iki cyatuma uhinduka ukaba umukiriya wa sosiyete Inyenyeri mu gihe kiri imbere? |
|  |  |
| **FOR ALL HOUSEHOLDS IBIBAZO BIGENEWE IMIRYANGO YOSE** | |
| **Wrap up and closing Kwibukiranya ibyaganiriweho no gusoza** | |
| 4.1 | Is there anything else you would like to add? Ese hari ikindi wifuza kongeraho ku byo twaganiriye? |
| 4.2 | Do you have any questions for me? Ese hari ikintu wifuza kumbaza? |

Thank you for talking with us today. Urakoze kutuganiriza uyu munsi.
